# Investigating *Salmonella* Eko from Various Sources in Nigeria by Whole Genome Sequencing to Identify the Source of Human Infections

**DOI:** 10.1371/journal.pone.0156212

**Published:** 2016-05-26

**Authors:** Pimlapas Leekitcharoenphon, Ibrahim Raufu, Mette T. Nielsen, Birthe S. Rosenqvist Lund, James A. Ameh, Abdul G. Ambali, Gitte Sørensen, Simon Le Hello, Frank M. Aarestrup, Rene S. Hendriksen

**Affiliations:** 1 National Food Institute, Technical University of Denmark, WHO Collaborating Center for Antimicrobial Resistance in Foodborne Pathogens and European Union Reference Laboratory for Antimicrobial Resistance, Kgs. Lyngby, Denmark; 2 Faculty of Veterinary Medicine, Department of Veterinary Microbiology, University of Ilorin, Ilorin, Nigeria; 3 National Food Institute, Technical University of Denmark, Søborg, Denmark; 4 Faculty of Veterinary Medicine, Department of Veterinary Microbiology and Parasitology, University of Abuja, Abuja, Nigeria; 5 Institut Pasteur, Unité des Bactéries Pathogènes Entériques, Centre National de Référence des Salmonella, Paris, France; Institut National de la Recherche Agronomique, FRANCE

## Abstract

Twenty-six *Salmonella enterica* serovar Eko isolated from various sources in Nigeria were investigated by whole genome sequencing to identify the source of human infections. Diversity among the isolates was observed and camel and cattle were identified as the primary reservoirs and the most likely source of the human infections.

## Introduction

Worldwide, *Salmonella* is estimated to cause 93.8 million human infections and 155,000 deaths annually [1]. Various production animals including poultry, pigs, and cattle are reservoirs for *Salmonella*, but reptiles also seem to play a role infecting people in sub-Saharan Africa [2]. In most developed countries, *Salmonella enterica* serovars Typhimurium and Enteritidis are the major reported causes of human salmonellosis [3]. However, other serovars, some rarely described, are common in specific geographical regions such as in Nigeria [2,4,5]. Recently, the first attempt for an active One-Health laboratory-based *Salmonella* surveillance program targeting both humans and animals was launched in the north-eastern region of Nigeria [6].

A total of 1,888 samples were collected from various sources in Nigeria from 2009 to 2011 [6]. Of those, 149 samples were found positive for up to 17 different *Salmonella* serovars and *S*. Eko dominated and was found in 26 of the samples; 12 samples from cattle, 7 from camels, 6 from human feces, and 1 sample from fish. The isolates were previously serotyped followed by minimum inhibitory concentration (MIC) determination as per Ibrahim et al. [6].

*S*. Eko is rarely reported and has to our knowledge only been reported from poultry sources in Cameroun in 2006–2007 [7].

The purpose of this investigation was to obtain more knowledge about the diversity of *S*. Eko and determine whether humans were infected with *S*. Eko from cattle, camels or fish.

## Materials and Methods

Genomic DNA was extracted from the 26 isolates, including the original *S*. Eko serovar reference strain #1279 isolated from a patient in the city of Eko, Nigeria, in 1973, using an Invitrogen Easy-DNA^™^ Kit (Invitrogen, Carlsbad, CA, USA), and DNA concentrations were determined using the Qubit dsDNA BR assay kit (Invitrogen). The genomic DNA was prepared for Illumina pair-end sequencing using the Illumina (Illumina, Inc., San Diego, CA) NexteraXT^®^ Guide 150319425031942 following the protocol revision C (http://support.illumina.com/downloads/nextera_xt_sample_preparation_guide_15031942.html). A sample of the pooled NexteraXT libraries was loaded onto an Illumina MiSeq reagent cartridge using MiSeq Reagent Kit v2 and 500 cycles with a standard flow cell. The libraries were sequenced using an Illumina platform and MiSeq control software 2.3.0.3. All isolates were pair-end sequenced with 2 x 150bp base reads.

Raw sequence data were submitted to the European Nucleotide Archive (http://www.ebi.ac.uk/ena) under study accession no.: PRJEB13610 (http://www.ebi.ac.uk/ena/data/view/PRJEB13610). The raw reads were assembled using the Assemble pipeline (version 1.0) available from the Center for Genomic Epidemiology (CGE) http://cge.cbs.dtu.dk/services/all.php, which is based on the Velvet algorithms for *de novo* short reads assembly. A complete list of genomic sequence data is available in the S1 Table. The assembled sequences were analyzed to identify the MLST sequence type (ST) for *Salmonella enterica* based on the publication by Jolley KA *et al*., 2010 [8]. In addition, plasmid replicons, pMLST, and acquired antimicrobial resistance genes using the pipelines MLST (version 1.7), PlasmidFinder (version 1.2), pMLST (version 1.4), and ResFinder (version 2.1) available from CGE [9–11]. Single Nucleotide Polymorphisms (SNPs) were determined using the pipeline CSI Phylogeny (version 1.1) available from the CGE website [12]. Basically, each of the raw reads was aligned against the genome of strain #2 due to the lack an appropriate reference genome, using Burrows-Wheeler Aligner (BWA) version 0.7.2 [13]. SNPs were called using ‘mpileup’ module in SAMTools version 0.1.18 [14]. SNPs were selected based on both coding and non-coding regions and when they met the following criteria: 1) a minimum distance of 15 bps between each SNP (e.g. to exclude homopolymer-rich regions), 2) a minimum of 10% of the average depth, 3) the mapping quality was above 30, 4) the SNP quality was more than 20 and 5) all indels were excluded.

The qualified SNPs from each genome were concatenated to a single alignment corresponding to position of the strain using an in-house Perl script. In case SNPs were absent in the genome of strain #2, they were interpreted as not being a variation and the relatively base from the genome of strain #2 was expected [15]. The concatenated sequences were subjected to multiple alignments using MUSCLE from MEGA5 [16]. The final phylogenetic SNP tree was computed by MEGA5 using the maximum likelihood method of 1,000 bootstrap replicates [17] using the Tamura-Nei model for inference [18].

## Results

The MLST showed that all strains except for #53 (cattle) shared the same loci and alleles; *aroC*-17, *dnaN*-101, *hemD*-8, *hisD*-439, *purE*-6, *sucA*-117, and *thrA*-12; sequence type (ST)2979. Isolate #53 was a single locus variant with an alteration in allele *aroC*-114; ST2980. Interestingly, the original *S*. Eko reference strain #1279 from1973 also displayed a completely different and unknown MLST, *aroC*-426, *dnaN*-148, *hemD*-18, *hisD*-43, *purE*-140, *sucA*-127, *thrA*-48.

**Fig 1:**
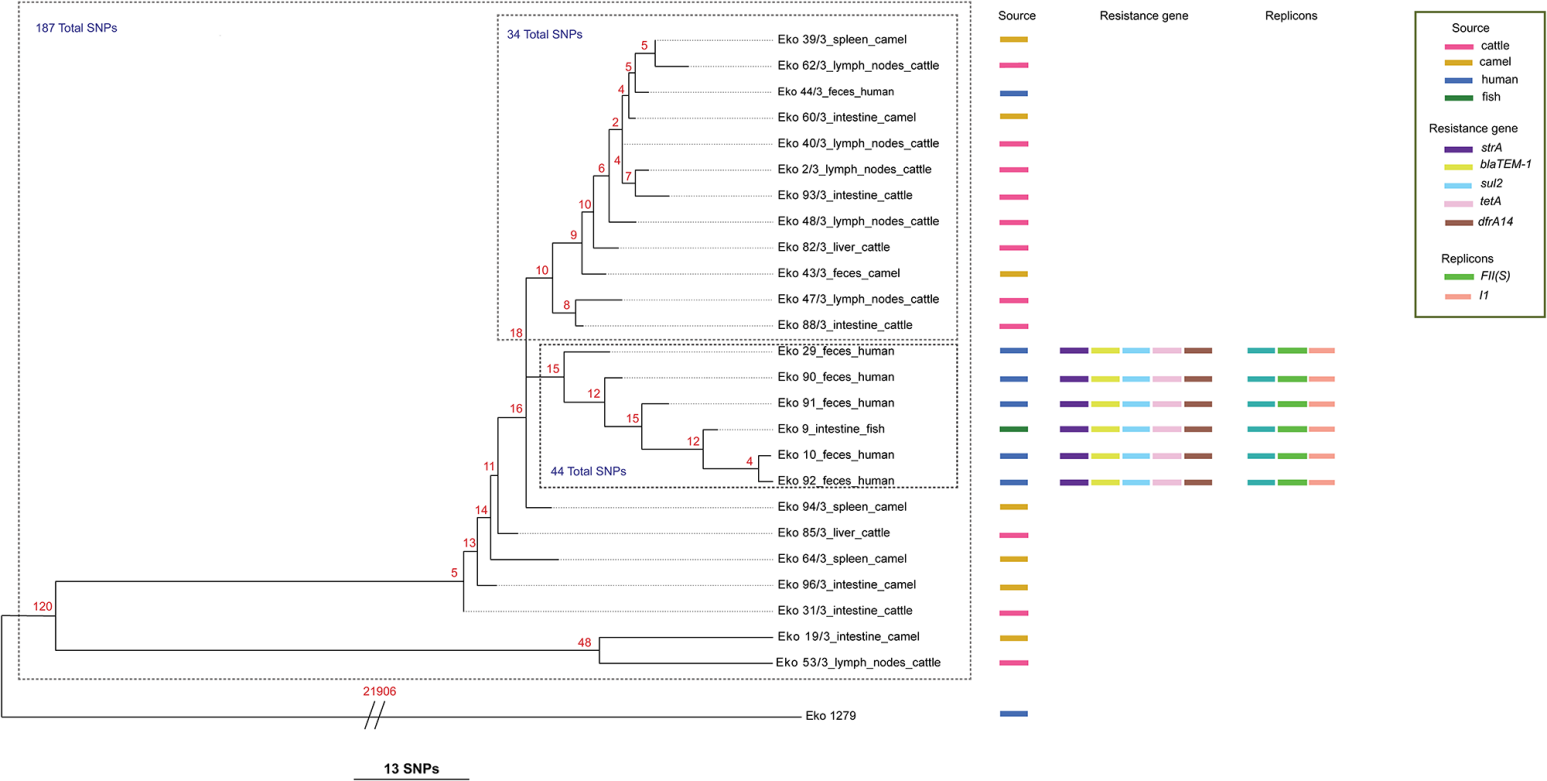
Phylogenetic reconstruction of the genetic relationships among the *S*. Eko isolates from cattle, camel, humans and fish in the north-eastern region of Nigeria.

Six of the isolates originating from human, #10, #29, #90, #91, # 92, and fish, #9, contained plasmid replicons; *inc*FII and *inc*I1 (Fig 1). The pMLST showed that *inc*FII exhibited one plasmid multilocus allele FII-S3 and sequence type as S3:A-:B- and *inc*I1 contained a plasmid multilocus profile (*ardA*-1, *pill*-6, *sogS*-7, *repI*1-5, *trbA*-3) (official nomenclature).

The MIC determination and detection of antimicrobial resistance genes showed that the same six isolates shared the same plasmid replicons, antimicrobial resistance genes, and conferred resistance to the same antimicrobials. They were all resistant to and harbored the following resistance genes: trimethoprim (TMP) *dfrA14*; streptomycin (STR) *str*A; tetracyclin (TET) *tet*A; sulfamethoxazole (SMX) *sul*2; and ampicillin (AMP) *bla*_TEM-1b_. The remaining part of the strains was pan-susceptible and did not harbor any plasmid replicons (Table 1 and Fig 1).

**Table 1:** MIC determination and antimicrobial resistance genes of *S*. Eko isolates from different sources in the north-eastern region of Nigeria.

Sample no.	Origin	Resistance breakpoints (μg/ml) and percent similarity of resistance genes									
		AMP R>8	*blaTEM-1*	STR R>16	*strA*	SMX R>256	*sul2*	TET R>8	*tetA*	TMP R>2	*dfrA14*
2/3	Cattle/lymph nodes	2	-	16	-	512	-	< = 2	-	< = 1	-
40/3	Cattle/lymph nodes	2	-	16	-	256	-	< = 2	-	< = 1	-
47/3	Cattle/lymph nodes	2	-	16	-	256	-	4	-	2	-
48/3	Cattle/lymph nodes	2	-	16	-	< = 64	-	< = 2	-	< = 1	-
53/3	Cattle/lymph nodes	2	-	16	-	< = 64	-	< = 2	-	< = 1	-
62/3	Cattle/lymph nodes	< = 1	-	16	-	< = 64	-	< = 2	-	< = 1	-
31/3	Cattle/ intestine	2	-	32	-	256	-	< = 2	-	< = 1	-
88/3	Cattle/ intestine	2	-	16	-	128	-	< = 2	-	< = 1	-
93/3	Cattle/ intestine	2	-	< = 8	-	< = 64	-	< = 2	-	< = 1	-
82/3	Cattle/liver	2	-	16	-	256	-	< = 2	-	< = 1	-
85/3	Cattle/liver	< = 1	-	< = 8	-	512	-	< = 2	-	< = 1	-
19/3	Camel/ intestine	2	-	16	-	256	-	< = 2	-	< = 1	-
60/3	Camel/ intestine	< = 1	-	16	-	< = 64	-	< = 2	-	< = 1	-
96/3	Camel/ intestine	2	-	< = 8	-	128	-	< = 2	-	< = 1	-
43/3	Camel/feces	2	-	16	-	512	-	< = 2	-	< = 1	-
39/3	Camel/spleen	2	-	16	-	256	-	< = 2	-	< = 1	-
64/3	Camel/spleen	2	-	< = 8	-	128	-	< = 2	-	< = 1	-
94/3	Camel/spleen	< = 1	-	< = 8	-	256	-	< = 2	-	< = 1	-
9	Fish/intestine	>32	+	16	+	>1024	+	>32	+	> = 32	+
10	Human/feces	>32	+	16	+	>1024	+	>32	+	> = 32	+
29	Human/feces	>32	+	16	+	>1024	+	>32	+	> = 32	+
44/3	Human/feces	4	-	32	-	256	-	< = 2	-	< = 1	-
90	Human/feces	>32	+	16	+	>1024	+	>32	+	> = 32	+
91	Human/feces	>32	+	16	+	>1024	+	>32	+	> = 32	+
92	Human/feces	> = 32	+	16	+	> = 1024	+	> = 32	+	> = 32	+

MIC (μg/mL) determined in accordance with CLSI standards. Abbreviations: +, presences of the resistance gene; AMP, ampicillin; STR, streptomycin; SMX, sulfamethoxazole; TET, tetracyclin; TMP, trimethoprim. The isolates were susceptible to the following antimicrobial agents: AMC, amoxicillin-clavulanic acid; APR, apramycin; CHL, chloramphenicol; CIP, ciprofloxacin; COL, colistin; CTX, cefotaxime; FFN, florfenicol; GEN, gentamicin; NAL, nalidixic acid; NEO, neomycin; SPT, spectinomycin; XNL, ceftiofur.

The genetic relatedness of the 26 *S*. Eko isolates was examined using a phylogenetic SNP analysis that identified 22,080 high-quality whole genome SNPs among the genomes. The high number of SNPs was a result of including the original serovar reference strain isolated in 1973, which was separated by an average of 21,906 SNPs and a standard deviation of 14 SNPs from the other genomes included in this study.

The topology of the phylogenetic tree based on the 25 contemporary isolates revealed a total of 187 SNPs and formed 2 distinct clusters; 1 cluster of the 6 genomes including the 5 human and 1 fish isolate, and another cluster of 12 genomes containing 8 cattle, 3 camel and 1 human isolate. The cluster formed by the 5 human and 1 fish isolate was genetically linked, separating the individual genomes between 4 to 15 SNPs. The remaining seven isolates formed single individual branches.

## Discussion

The strains belonged to more than one MLST as a result of a single nucleotide variation and the field strains were separated by 187 SNPs which argue for *S*. Eko being a monomorphic serovar similarly to *S*. Typhi [19]. However, the huge divergence of the current cluster of field isolates in comparison with the historical reference strain from 1973 and that this strain exhibited nucleotide variation in all seven allele rather arguing for a polymorphic serovar [20]. Thus, only if artificial divergence issuing from multiple laboratory sub-cultivations of the 1973 strain can be eliminated. To truly define *S*. Eko as an either mono- or polymorphic serovar will require additional analysis of more spatial and temporal strains which unfortunately are not available.

The likely reason why the fish intestine contained a resistant strain compared with the cattle and camel is that cattle and camels in most cases are grazing in the fields with limited veterinary attention and no antimicrobial treatment, whereas, the fish (intestine) might be contaminated from domestic, industrial and agricultural discharges (runoff water). Thus, fish and other aquatic life forms are vulnerable to all environmental hazards why the *Salmonella* could have originated from terrestrial sources such as the unorthodox utilization of poultry feces as manure/fertilizer on farmland located close to the river, consequently, during the rainy season the top-soil is washed into the river/pond leading to environmental and food (fish) contamination. Other factors such as poor sewage disposal coupled with a high water table permits untreated sewage to enter lakes or ponds either through runoff or storm-water.

A few of the isolates, #2, #85 and #43, were phenotypically resistant to sulfamethoxazole with one MIC value above the breakpoint, but they lacked the corresponding resistance genes. There was a similar observation for strains #31 and #44 for streptomycin. This phenomenon with borderline streptomycin resistant isolates, is well-documented and resistance should be ignored with the absence of corresponding resistance genes [21].

Usually non-typhoid *S*. *enterica* are found among mammals such as pigs, cattle and poultry [22] and rarely fish [23]. However, it is likely that the fish was incriminated by contamination from runoff or storm-water. Thus, the authors believe that fish was not the primary reservoir of the pathogen despite the similarity.

The genomes forming the cluster containing pan-susceptible isolates of human, camel and primarily cattle origins were only separated by two to ten SNPs. Thus, suggesting some of the genomes are clonal and that camel and cattle are the primary reservoirs of *S*. Eko and likely responsible for infecting the patient of this study. The study did not take into account for local genome rearrangements (double recombination events) which potentially also could lead to the differences observed conducting the SNP analysis.

In conclusion, this study points to cattle and camels as the primary reservoir of *S*. Eko and source of human infections. We encourage the public health authorities in Nigeria to support surveillance initiatives of zoonotic and antimicrobial resistance to further investigate the burden of these diseases and set up prevention measurements.

## Supporting Information

S1 Table (XLSX)
